# Natural language processing in clinical neuroscience and psychiatry: A review

**DOI:** 10.3389/fpsyt.2022.946387

**Published:** 2022-09-14

**Authors:** Claudio Crema, Giuseppe Attardi, Daniele Sartiano, Alberto Redolfi

**Affiliations:** ^1^Laboratory of Neuroinformatics, IRCCS Istituto Centro San Giovanni di Dio Fatebenefratelli, Brescia, Italy; ^2^Department of Informatics, University of Pisa, Pisa, Italy; ^3^Istituto di Informatica e Telematica, Consiglio Nazionale delle Ricerche, Pisa, Italy

**Keywords:** natural language processing, information extraction, deep learning, electronic health record, neuroscience, psychiatry

## Abstract

Natural language processing (NLP) is rapidly becoming an important topic in the medical community. The ability to automatically analyze any type of medical document could be the key factor to fully exploit the data it contains. Cutting-edge artificial intelligence (AI) architectures, particularly machine learning and deep learning, have begun to be applied to this topic and have yielded promising results. We conducted a literature search for 1,024 papers that used NLP technology in neuroscience and psychiatry from 2010 to early 2022. After a selection process, 115 papers were evaluated. Each publication was classified into one of three categories: information extraction, classification, and data inference. Automated understanding of clinical reports in electronic health records has the potential to improve healthcare delivery. Overall, the performance of NLP applications is high, with an average F1-score and AUC above 85%. We also derived a composite measure in the form of Z-scores to better compare the performance of NLP models and their different classes as a whole. No statistical differences were found in the unbiased comparison. Strong asymmetry between English and non-English models, difficulty in obtaining high-quality annotated data, and train biases causing low generalizability are the main limitations. This review suggests that NLP could be an effective tool to help clinicians gain insights from medical reports, clinical research forms, and more, making NLP an effective tool to improve the quality of healthcare services.

## Introduction

In recent years, natural language processing (NLP) has become an increasingly important topic in the artificial intelligence (AI) landscape of medical community. NLP is, intuitively, an ensemble of techniques to automatically process human language. It includes various tasks that can be divided into two main groups: the first group is represented by techniques that extract insights from a text (e.g., data extraction from medical reports, patient satisfaction understanding, sentiment analysis for drug evaluations), while the second group is related to text generation (e.g., translation, summarization, automatic chatbots). The rise in popularity of NLP in the last decade is due to several factors. First, the pervasive diffusion of the Internet has made it possible to access vast amounts of human-written text with little effort. For example, one of the currently most popular NLP language models, called bidirectional encoder representations from transformers (BERT) (1), has been pre-trained using Wikipedia texts and the Book corpus. Moreover, social media users produce an enormous amount of text every day; this source of information can be used for a variety of NLP tasks (2, 3). Another reason for the explosion in popularity of NLP is technological: in the last years, major improvements in neural networks (NNs) and deep learning (DL) architectures have enabled the transition from statistical to more complex and flexible models. One of the latest improvements in NLP is the introduction of Transformers, an architecture that has broken several records in NLP tasks (4). Moreover, NLP is strongly promoted by the world’s leading companies (e.g., Google, Amazon, OpenAI), which make a large part of their tools publicly available and open-source, increasing the general interest in NLP.

**FIGURE 1:**
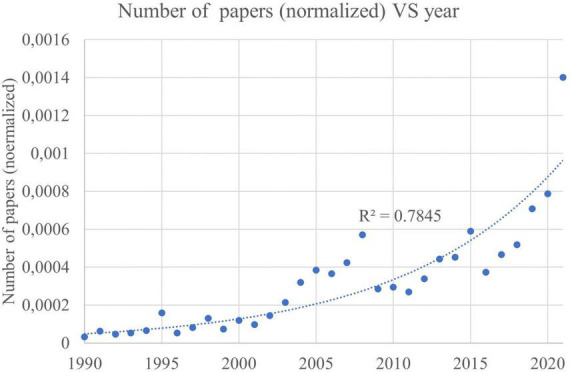
Number of scientific papers with keyword “Natural Language Processing” published on PubMed from 1990 to 2021, normalized on the number of total papers published on PubMed.

In the last decade, NLP improved significantly, thanks to the breakthroughs represented by word embeddings (5), Attention mechanism, and Transformers. However, its applications to neuroscience and psychiatry are still in their infancy. The ever-growing interest in NLP applications is reflected in the number of scientific papers on this subject. As can be seen in Figure 1, NLP-related papers show an exponential-like trend (*R*^2^ = 0.78). In 1990 there were 13, in 2000 59, in 2010 242, and in 2021 already 1,618. The medical field is one of the areas that could benefit most from solid and reliable NLP models, mainly because most of the medical texts produced daily in hospitals and clinics are unstructured and not exploited as they could be. NLP can be used effectively for various medical tasks, such as earlier diagnosis, better identification of candidates for medical procedures, or to assist physicians in their decision-making. The purpose of this review is to explore how NLP can be applied to neuroscience/psychiatric clinical documents to help clinicians gain insights from them.

### Related work

The rationale of this review is to provide an overview of the applications of NLP in neuroscience and psychiatry. The target audience is primarily clinicians working in these fields, who could benefit from both the comprehensive yet simple overview of the state-of-the-art of NLP, and the possible declinations of these technologies in real case scenarios.

As reported in Section “Methods,” other NLP reviews were also analyzed. Due to their nature, they were not categorized into any of the three topics presented in Section “Results.” Some of these reviews are extremely focused on a specific argument (6–10); this makes them good candidates for topic-specific analysis, but they lack a general vision. Other reviews do not present the breakthroughs of the last 4–5 years (attention mechanism, transformers, BERT-models, etc.), either because they are relatively old (11–13), or because they present the current state-of-the-art at a high level (14–17). Lastly, Wu et al. (18) present a solid review, which is focused on DL approaches only. For these reasons, we believe that the current review can be of interest to the target audience: it provides a broad overview of the current NLP landscape, both in terms of technical approaches and clinical applications, and it also presents works that apply a traditional machine learning (ML) approach (i.e., algorithms that do not involve NNs), thus providing a complete picture of the current state-of-the art of NLP applied to neuroscience and psychiatry.

The paper is structured as follows: first, an overview of NLP is presented with a formal definition (Box 1), a brief history, and an explanation of the main tools available. Then, follows a paragraph on the selection method of the papers presented in this work. This is followed by a paragraph on the three major NLP topics [i.e., information extraction (IE), classification, data inference]. Discussion and conclusions are then drawn. A glossary of some of the terms used in this review can be found in Box 2.

**BOX 1 Natural Language Processing Definition.**

NLP is “*a theoretically motivated range of computational techniques for analyzing and representing naturally occurring texts at one or more levels of linguistic analysis for the purpose of achieving human-like language processing for a range of tasks or applications”* (115).

NLP is *“an area of research and application that explores how computers can be used to understand and manipulate natural language text to do useful things*” (116).

**BOX 2 Glossary of all the NLP terms used in the review.**

**Annotation:** see Labeling.

**Attention:** mechanism that enables the model to focus on important parts of the context, no matter how distant from the current word;

**Augmentation strategy:** techniques used to generate additional, synthetic data;

**Corpus (plural corpora)**: a collection of written texts. They can be structured or not, annotated or not;

**Entity Linking:** the operation of assigning logical relationships between Named Entities;

**Fine-tuning**: process of further training a pre-trained model on a specific task by means of labeled data;

**Labeling:** the process of assigning values to raw data;

**Part-of-speech tagging**: assigning word types to tokens (e.g., verb, noun);

**Pre-training**: process of training a model with unlabeled data, thus giving it a general understanding of the subject;

**Stemming:** reducing a word to its base root by cutting off its end (or beginning);

**Transformer:** model architecture that relies entirely on the attention mechanism to draw global dependencies between input and output;

**Transfer learning:** machine learning technique where a model developed for a task is reused as the starting point for a model on a second task;

**Tokenizer:** component of the NLP pipeline that performs tokenization, i.e., splitting a text into smaller units called tokens (words, subwords, or characters):

## Overview of natural language processing architectures

From a practical point of view, NLP is a class of algorithms that process text expressed in natural language to achieve a goal (see Box 3 and Box 4). When we talk about architectures, we can distinguish a pre-NN era and an NN era. The pre-NN era started in the late 1940s, with the primary task under study being machine translation (19). Various approaches were developed over the years, from grammar theories in the 1960s (20) to symbolic approaches in the 1980s (21). In the early 1990s, systems based on hand-written rules were abandoned in favor of statistical models (22), thanks to the increasing computing power and the advent of ML algorithms. NLP got a spike of interest in the last decade, thanks largely to advances in DL and the availability of human-written texts.

**BOX 3 NLP Extractive tasks - Objective: gain insight from texts.**

**Named Entity Recognition (NER):** identifies words in a text belonging to predefined semantic types, e.g., person, location (117). NER often serves as the foundation for many other NLP tasks;

**Relation extraction:** extracts semantic relationships (associations between the meanings of words) connecting two specified named entities in a sentence (118);

**Question Answering** (QA): inputs are a text (context) and a question. Its goal is to find the span in the context that answers the question, if possible. Most advanced QA systems can also understand if the question has no answer (119);

**Topic modeling:** also called *topic analysis*, its goal is extracting the main topics that occur in a text (120);

**Text similarity:** also called *semantic similarity*, its goal is to produce a score that expresses how similar the meanings of texts are (121);

**Sentiment analysis:** usually performed on reviews, produces an output that tells if the text expresses a positive, neutral, or negative sentiment (122).

**BOX 4 NLP Generative tasks–Objective: generate a text starting from another.**

**Translation:** the first task ever studied in NLP; it is the operation of translating a text from a language to another;

**Summarization:** its goal is to make a text shorter while preserving most of the essential information;

**Text generation:** generically, it is the task of producing a plausible and readable text in human language from input data. This can be exploited in interactive chat bots, where a text is generated as a response from the user input, or for data augmentation.

### Neural network models

The era of NN models, also called large language models (LLMs) when applied to NLP, started at the beginning of the new millenium, when the first neural language model was created (21). Since then, several NN approaches have appeared in NLP research (23). In 2010, the model semantic/syntactic extraction using a neural network architecture (SENNA) was implemented. It was the first word embedding architecture able to perform several NLP tasks (5). A few years later, the Word2Vec model was presented (24): the authors trained an NN on very large unannotated corpora, showing the power of using pre-trained embeddings. However, Word2Vec has limitations: most importantly, it does not take into account the context of the words. For example, the word “bank” would have exactly the same representation as “bank-deposit” and “river-bank.”

In the following years, some popular DL architectures started to be used in the field of NLP: convolutional neural networks (CNNs) (25), fast and highly parallelizable, and recurrent neural networks (RNNs), which are ideal for sequential data such as texts. CNNs, which have been used extensively in computer vision, can also be applied to common NLP tasks, such as text classification (26). Since texts are sequences of words, RNNs are suitable for NLP applications (27), because they examine each element of a sequence once and retain it in memory for reuse when examining the next elements. However, standard RNNs have difficulty learning long-term temporal dependencies. The problem has been solved by the introduction of long short-term memory networks (LSTM) (28). LSTMs are a special type of RNNs that are able to retain information in memory over long periods of time.

Recently, the NLP scene has been dominated by transformers, which are based on the attention mechanism (4). Compared to previous DL architectures, these models proved to be qualitatively superior while being more parallelizable, requiring significantly less time to train. Transformers paved the way for the development of the aforementioned BERT (1), developed by Google. BERT, which generates representations of words based on their context, boosted the performance of various NLP tasks and established itself as state-of-the-art (29). Since then, several BERT-based architectures have been developed: robustly optimized BERT approach (RoBERTa) (30), and a lite BERT (ALBERT) (31) are among the most known. Another reason for the success of these architectures is transfer learning (TL), a technique that allows an already pre-trained model to be used and specialized with much less labeled data. This operation is called fine-tuning.

Nowadays, hundreds of transformer models are available in the NLP landscape, and most of them perform better than BERT. While it is virtually impossible to keep track of every single one, the most significant are here presented:

1. XLNet (32), which requires a huge amount of data to train and very high computational power;
2. Generative pre-trained transformer (GPT) (33), whose latest version 3 was released in summer 2020. The model has been developed in collaboration with Microsoft and is not open-source;
3. Text-to-text transfer transformer (T5) (34), an encoder-decoder model pre-trained on a multi-task mixture of unsupervised and supervised tasks and for which each task is converted into a text-to-text format;
4. Wenxin,^1^ the world’s first knowledge-enhanced 100-billion-scale pre-trained language model and largest Chinese-language monolithic model;
5. Megatron-Turing (35), developed by Microsoft and nVIDIA, the largest and the most powerful monolithic transformer language model trained to date, with 530 billion parameters (3x GPT-3).

### Bidirectional encoder representations from transformers and bidirectional encoder representations from transformers-based models

BERT uses a WordPiece tokenizer (36) (i.e., words are split into subwords before being processed), and its pre-training is based on two principles: the masked language modeling (MLM) and the next sentence prediction (NSP). MLM randomly masks 15% of the tokens with a [MASK], and the model tries to predict the original ones based on the surrounding words. In this way, the representation can merge the left and right contexts, making the model deeply bidirectional. In the NSP task, the model is given pairs of sentences and learns to predict whether they follow each other. BERT has broken records in several NLP tasks and has become the *de facto* reference model for NLP. Moreover, its popularity is based on the possibility of fine-tuning a model to a specific task by adding further layers that are trained on task specific data of smaller size, which significantly reduces the time and resources needed to build customized models.

Although effectiveness has been achieved in various NLP tasks, BERT has difficulties when dealing with very specific contexts. To address this problem, researchers have developed more focused models, starting from the original BERT. As far as the biomedical domain is concerned, three of the most popular BERT-based models are bidirectional encoder representations from transformers for biomedical text mining (BioBERT) (37), Clinical (Bio)BERT (38), and unified medical language system-bidirectional encoder representations from transformers (Umls-BERT) (39).

#### Bidirectional encoder representations from transformers for biomedical text mining

BioBERT is a domain-specific language representation model, pre-trained on large biomedical corpora (PubMed abstracts and full-text articles). BioBERT outperforms BERT and previous state-of-the-art models in several biomedical text mining tasks performed on biomedical corpora: named entity recognition (NER) (the task of identifying words in a text that belong to predefined semantic types), biomedical relation extraction, question answering (QA).

#### Clinical (Bio)BERT

Clinical BERT and Clinical BioBERT are models fine-tuned on medical corpora, initialized from BERT-Base and BioBERT, respectively. They were tested with different NLP tasks (i.e., MedNLI (40), i2b2 2006,^2^ 2010, 2012, and 2014). In three out of the five tasks, they showed improvements over both BERT and BioBERT. These results demonstrate the utility of using domain-specific contextual embeddings for biomedical NLP tasks.

#### Unified medical language system-bidirectional encoder representations from transformers

Umls-BERT was motivated by the fact that BioBERT and Clinical BERT do not take into consideration structured expert domain knowledge from a knowledge base. Umls-BERT integrates domain knowledge during pre-training by means of an augmentation strategy. This is done by connecting words that have the same underlying concept in unified medical language system (UMLS) and leveraging semantic type knowledge in UMLS to create clinically meaningful input embeddings. Umls-BERT outperforms existing domain-specific models on common NER and clinical natural language inference tasks.

### Natural language processing generic tools

The research community has developed numerous tools for performing NLP tasks. Most are open-source libraries that allow users with basic programming skills to perform language analysis. We present three of the most popular open-source libraries for NLP: natural language toolkit (NLTK),^3^ spaCy,^4^ and hugging face (HF) (41).

#### Natural language toolkit

Natural language toolkit is a platform for developing software to work with natural language, originally developed in 2001 as part of a computational linguistics course at the University of Pennsylvania. It provides a suite of text processing libraries for classification, tokenization, stemming (i.e., reducing a word to its base root), part-of-speech tagging (i.e., assigning word types to tokens, such as verb or noun), parsing, and more. NLTK covers symbolic and statistical NLP and is easy to use, making it suitable for linguists, researchers, and industrial users. The main limitation of NLTK is that it does not implement modern NN and DL models.

#### SpaCy

SpaCy is an open-source library for advanced NLP in Python, designed specifically for production use. It can be used to build natural language understanding systems, or to pre-process texts for DL architectures. SpaCy, unlike NLTK, is not a research software. This means that the user will not be asked how to implement a pipeline: this is done automatically, ensuring the highest possible execution speed. DL models are provided in the library.

#### Hugging face

Hugging face is a company that develops open-source resources to easily integrate AI into workflows. In particular, they have developed the Transformers library, which is designed to handle Transformer architectures. HF has a very large and active community and provides thousands of pre-trained and fine-tuned models, some of which have been developed by the world’s leading companies (e.g., Google, Microsoft) and allow to obtain state-of-the-art results on many NLP tasks. With the HF libraries users can perform many operations, such as creating personalized tokenizers, pre-training models from scratch, fine-tuning already existing models, and sharing them with the community.

### Natural language processing medical tools

Generic NLP tools are very popular in the scientific community. However, in the last decade, many medical-NLP tools have also become available, especially for the task of IE, NER, and entity linking (the operation of assigning logical relationships between named entities). In this section, the most popular ones are presented.

#### Clinical text analysis and knowledge extraction system

Clinical text analysis and knowledge extraction system (cTAKES) (42) is an NLP system for extracting information from electronic health records (EHRs) clinical free text. It can identify classes of clinical named entities such as drugs, diseases, and symptoms. Originally developed at the Mayo Clinic, it is now used by various institutions around the world.

#### Biomedical-yet another open data information extraction system

Biomedical-yet another open data information extraction system (Bio-YODIE) (43) is a named entity linking system for biomedical texts. Bio-YODIE implements a pipeline that applies annotations to documents containing a UMLS (44) Concept Unique Identifier along with other relevant information from the UMLS. Bio-YODIE shows results comparable to other named entity linking systems presented in the literature.

#### MetaMap

MetaMap (45) is a highly configurable program for mapping biomedical texts to the UMLS Meta-thesaurus. The development of MetaMap was guided by linguistic principles that provide both a rigorous foundation and a flexible architecture. It uses a knowledge-intensive approach based on symbolic NLP and computational-linguistic techniques. After tokenization, part-of-speech-tagging, and syntactic analysis, candidates are identified and then combined to produce a final result that best matches the text and the concept of the phrase. A lite version of MetaMap was developed to focus on real-time processing speed (46).

#### Medical concept annotation toolkit

Medical concept annotation toolkit (MedCAT) (47) is an open-source tool with many features: NER, annotation tool, and online learning training interface. Its annotation tool, MedCATtrainer, allows clinicians to review, improve and adapt extracted concepts via a web interface designed for training IE pipelines. To evaluate MedCAT, the following corpora were used: ShARe/CLEF (48) and MIMIC-III (49) datasets. MedCat outperformed other popular medical NLP tools (e.g., cTAKES, Bio-YODIE, MetaMap) in almost all aspects and proved to be an effective tool for specific analyses.

Table 1 gives a summary of the NLP tools and resources that were utilized in the included studies. Table 2 include some additional details.

**TABLE 1:** Qualitative evaluation of BERT models, generic NLP and medical NLP tools.

		Availability of materials (e.g., pre-trained models, ontologies, corpora, etc.)	Possibility of customization	Ability to perform basic NLP tasks (e.g., tokenization, stemming, POS tagging, etc.)	Ability to perform advanced NLP tasks (e.g., QABot, summarization, etc.)	Usability of APIs for the analysis of medical data	Community activeness	Scalability/User-friendliness
BERT models	BioBERT	Good^1^	Good	†	†	Excellent	Average	Average
	Clinical BioBERT	Good^1^	Good	†	†	Excellent	Poor	Poor
	Umls-BERT	Good^1^	Good	†	†	Excellent	Poor	Poor
NLP generic tools	NLTK	Good	Excellent	Excellent	None	None	Excellent	Excellent
	spaCy	Excellent	Good	Excellent	Good	Average	Excellent	Excellent
	HF	Excellent	Excellent	Excellent	Excellent	Average	Excellent	Excellent
NLP medical tools	cTAKES	Good	Good	Excellent	None	Excellent	Good	Average
	Bio-YODIE	Average	Average	Good	None	Excellent	Poor	Poor
	MetaMap	Average	Average	Good	None	Excellent	Average	Average
	MedCAT	Average	Average	Good	None	Excellent	Excellent	Good

The rating scale is (from worst to best): None, Poor, Average, Good, and Excellent. NLP, natural language processing; BERT, bidirectional encoder representations from transformers; BioBERT, bidirectional encoder representations from transformers for biomedical text mining; NLTK, natural language toolkit; HF, hugging face; cTAKES, clinical text analysis and knowledge extraction system; Bio-YODIE, biomedical-yet another open data information extraction system; MedCAT, medical concept annotation toolkit. ^1^Model is free to download. ^†^It is not possible to evaluate BioBERT, Clinical BioBERT, and Umls-BERT ability to perform NLP tasks a priori because they require fine-tuning first.

**TABLE 2:** Natural language processing (NLP) tools and web sites.

NLP tools	Brief description	Web-site
NLTK	Open-source library for low-level NLP operations (stemming, part-of-speech tagging)	https://www.nltk.org/
spaCy	Open-source library for advanced NLP in Python. Support 64+ languages, implement DL models	https://spacy.io/
Hugging face	Open-source library for NLP in Python. Features thousands of pre-trained models and datasets in several languages	https://huggingface.co/
cTAKES	Open-source NLP tool for IE from clinical EHR unstructured text	https://ctakes.apache.org/
Bio-YODIE	Open-source NLP tool for biomedical named entity linking pipeline	https://github.com/GateNLP/Bio-YODIE
MetaMap	Highly configurable program to map biomedical text to the UMLS Meta-thesaurus	https://lhncbc.nlm.nih.gov/ii/tools/MetaMap.html
MedCAT	Open-source NLP tool for IE from EHRs and link it to biomedical ontologies like SNOMED-CT and UMLS	https://github.com/CogStack/MedCAT

NLP, natural language processing; DL, deep learning; IE, information extraction; EHR, electronic health records; UMLS, unified medical language system; SNOMED-CT, systemized nomenclature of medicine-clinical terms.

## Methods

A search was conducted to identify all potentially relevant publications on NLP applications in neuroscience and psychiatry. We focused on publications in the time window from 2010 to early 2022. The ACL Anthology, PubMed, Embase, and PsycINFO repositories were queried in January 2022.

All publications that emerged from the search were independently assessed by two authors (CC, senior engineer with 24 months of experience in clinical neuroscience; AR, senior neuroscientist with 14 years of experience in neuroinformatics). Publications were excluded if full text could not be retrieved or if they were published in a journal without an assigned impact factor in the 2022 Journal Citation Report science edition.

**FIGURE 2:**
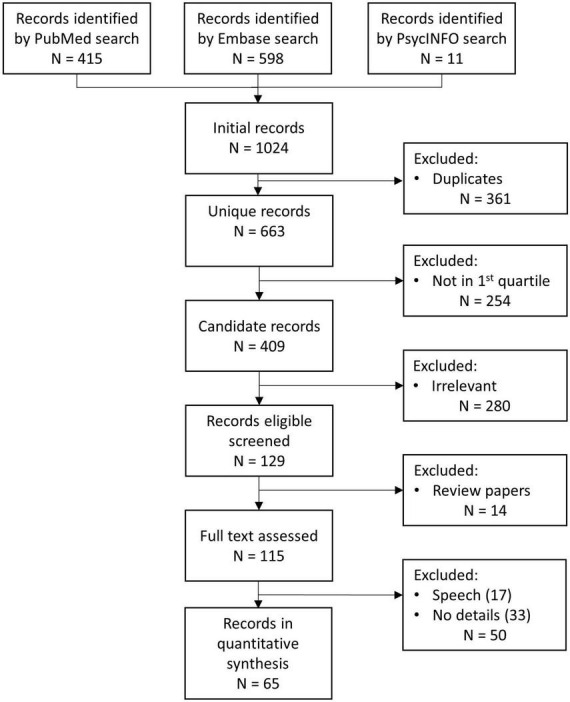
PRISMA chart.

The ACL Anthology database, which included about 77 thousand papers, was screened first. Articles published in Proceedings were removed to ensure a high-standard for the present review. This left 2,577 ACL Anthology papers, which became 884 after date filtering and deduplication. The remaining ACL Anthology articles were inspected but none was found to be related to neuroscience or psychiatry domains. Therefore, the ACL Anthology articles were not included in the PRISMA chart (Figure 2).

The subsequent search yielded a total of 415 publications from PubMed, 598 from Embase, and 11 from PsycINFO for a total of 1,024 initial records, which became 663 after deduplication. The search queries used to select records in PubMed, Embase, and PsycINFO are described in Box 5.

**BOX 5 Search strategy.**

PubMed: “[(Natural Language Processing) OR (NLP)) AND ((neurology) OR (psychiatry)] [‘2010’ (Date-Publication): ‘2022/01’ (Date-Publication)]”

Embase: “(‘natural language processing’/exp OR ‘natural language processing’) AND (‘neuroscience’/exp OR ‘neuroscience’ OR ‘psychiatry’/exp OR ‘psychiatry’)”

PsycINFO query 1: “Natural Language Processing AND Neuroscience”

PsycINFO query 2: “Natural Language Processing AND Psychiatry”

These papers were screened to determine whether they met the eligibility criteria. They were screened only if they were published in first quartile journals, in order to analyze high-quality articles. This process resulted in the exclusion of 254 publications, leaving 409 candidate records, that were divided into two groups: non-relevant and relevant articles. Articles that were not directly related to NLP topics or that were related to NLP but focused on biomedical topics unrelated to neuroscience or psychiatry were classified as non-relevant. A total of 280 articles were non-relevant. The remaining 129 relevant papers were screened, and 14 of them were excluded from the main analysis because they were reviews. Nevertheless, they were examined, and an overview is presented in Section “Related work.” The full texts of the remaining 115 papers were assessed: 17 of them were excluded because they focused on speech analysis, and 33 excluded because they did not contain sufficient information about the NLP algorithms used (Figure 2).

The remaining 65 papers were deemed appropriate and analyzed. Although they all relate to NLP, three distinct topics were identified:

1. Natural language processing for IE (*N* = 18): this class of articles implements (or applies an existing) system for IE to get insight into patients’ medical records;
2. Natural language processing for classification (*N* = 24): this class of articles describes works that exploit NLP-based features (e.g., identification and frequency of pathological words, number of different words used in the text, …) to train and deploy a classifier that predicts patient health status;
3. Natural language processing for outcome prediction (*N* = 23): this class of papers presents systems that use NLP-based tools to make assumptions and hypotheses regarding patients, e.g., to stratify potential candidates for a specific surgical operation.

## Results

### Natural language processing for information extraction

This class of papers focuses on IE. Some of them start from existing tools and extend them, while others use ML/DL architectures to develop new tools.

Creating a tool to perform IE in the biomedical domain from scratch is challenging. The process requires both a solid knowledge of modern NLP architectures and expertise in biomedical disciplines. While the first requirement is facilitated by the NLP open-source libraries, the second requires clinicians to make their skills and expertise available to NLP developers. This is because, even though TL has mitigated the impact of this issue, a large number of annotated texts are needed to properly train DL architectures. The annotation process, also known as labeling, necessarily needs someone knowledgeable in the medical field.

An overview of the creation process for an NLP-based biomedical tool is presented by Liao et al. (50). The first step is to define the main research objectives and the ideal study design and population. The following steps are to create a sensitive data repository, define a comprehensive list of potential variables and terms useful for the algorithm, create a training set, and develop the classification algorithm. The final step is the validation of the algorithm and statistical analysis of the results. The process is tedious, but the performance improvement that an NLP approach provides makes the effort worth.

This paradigm was also followed in the development of the aforementioned cTAKES, MedCAT, and ExECT (51). The ultimate goal was to create a general-purpose, open-source biomedical NLP tool that can be used for other applications; such tools can then be used to work on specific pathologies. This is the case of Johnson et al. (52), who used ClinicalRegex^5^ to examine social support in patients with aggressive hematologic malignancies. Khapre et al. (53) and McDonald et al. (54) similarly used General Architecture for Text Engineering (GATE),^6^ an open-source NLP toolkit, to study symptoms in women with severe mental illness (i.e., psychotic and bipolar disorders) and in people with mood instability and/or sleep disturbance, respectively.

The above tools have highlighted two major problems in the development of NLP tools, namely:

1. The vast majority of available systems is for the English language only;
2. One of the critical issues is the difficulty of having large annotated corpora in specific medical fields.

The first limitation can be overcome with a large corpus, as shown by Lopes et al. (55), who developed a Portuguese NER tool that achieves comparable performance to the English models. This drawback could be less severe with very large models, since they able to perform zero-shot learning (ZSL) (33), which means that a model can work on classes of data it has never seen before. In the NLP context, this allows models to perform fairly well even on tasks or languages that they have not been trained for. The second issue can be solved either manually, which implies a large annotation effort, or with a (semi)automatic approach, as shown by Yu et al. (56), who present an active learning system that allows the use of unannotated samples for training a NER pipeline.

Moving from general purpose biomedical NLP tools to more specialized ones, several works can be found in the neuroscience literature. Goodwin et al. (57), Pruitt et al. (58), and Choi et al. (59) used DL architectures to apply IE from electroencephalographic reports, cranial computed tomography scan reports, and pharmacokinetic and pharmacodynamic studies, respectively. NLP can also be used to study psychiatric disorders. As suggested by Palaniyappan (60) and Corcoran et al. (9), linguistic variables can be considered biomarkers of psychosis because they reflect biological processes. This assumption has been proven by Vaci et al. (61) and Mueller et al. (62), who developed algorithms to extract information from EHRs for depression and dementia, respectively.

Another subfield of IE is topic modeling, the goal of which is to discover the abstract topics in a text. This process can be performed both in both unsupervised and supervised ways. The former approach has the obvious advantage of eliminating the tedious annotation phase (63). The latter requires the development of a pipeline based on a supervised algorithm using tools, such as cTAKES (64). Conventional ML approaches can achieve comparable performance to DL models, but are more clinical interpretable, making them more understandable and acceptable in the clinical setting. This is not trivial: since such algorithms are used by clinicians as decision support tools, it is important for them to understand how decisions are made.

### Natural language processing for classification

The second class of the analyzed works refers to classification. Its goal is to create a model that performs classification starting from a text produced either by patients during a neuropsychological/psychiatric writing test (e.g., verbal fluency list) or by clinicians (e.g., medical notes). Such models typically exploit NLP features (e.g., frequency of words, number of adverbs, percentage of verbs in the past tense) along with standard features (e.g., vital parameters, psychological test scores) to train an AI classifier. Some of them are implemented using traditional ML models [e.g., support vector machine (SVM), random forest (RF)], others are based on DL architectures.

Texts created by patients generally follow certain patterns. By choosing the right features, models, and a large enough corpus, good results can be obtained using syntactic and semantic features to distinguish individuals with mild cognitive impairment from patients with Alzheimer’s disease or healthy controls from individuals with semantic dementia and progressive non-fluent aphasia (65, 66). In recent years, researchers have recognized that social medias can also be a source of text for these experiments. This is the case of Low et al. (67), Wang et al. (68), and Koh et al. (69): the first used posts from Reddit, the second and third from Twitter, to investigate how COVID-19 increased anxiety and loneliness in the population. Similarly, Howard et al. (70) exploited data from Reachout.com,^7^ an online mental health service for young people and their parents in Australia, benchmarking multiple methods of text feature representation for social media posts and comparing their downstream use with automated ML tools. Yu et al. (71) used data from the John Tung Foundation^8^ to build a framework for discovering linguistic association patterns, and showed that they are promising features for classification tasks.

As in the case of IE, the drawback is the lack of non-English tools. Indeed, there are few non-English NLP efforts in the literature that have collected large corpora to create *ad hoc* SVM classifiers for neuroscience domains that performed well in discriminating specific and non-specific memories from cue-recalled Japanese memories (72). However, in the non-English cases, the efforts required are colossal.

A different approach exploits notes written by clinicians (patient related notes or reports of instrumental examination). Some of these works uses NLP combined with traditional classifiers. Kim et al. (73), Li et al. (74), Castro et al. (75), Garg et al. (76), Xia et al. (77) exploited this approach using logistic regression, naïve Bayesian classifiers, RF, and SVM, respectively. Lineback et al. (78) used an XGBoost (79) and obtained similar results. Wissel et al. (80) implemented an NLP-based SVM application to assess eligibility for epilepsy surgery. As with IE, a similar approach can be applied to psychiatric disorders: Lin et al. (81), and Maguen et al. (82), implemented models to investigate unhealthy alcohol use and post-traumatic stress disorder, respectively. These works prove that NLP feature-based classifiers are feasible and yield good results, but they present limitations. First, the purpose of these tools is not to inform clinicians which feature makes the patient a good candidate, but to identify possible candidates: the models must be supplemented by medical assessments, which reinforces the idea that the final decision is always in the human hands. Another drawback could be the fact that all these models are trained on the results of one institution, which severely limit their flexibility. Results from the literature show that increasing the sample size by including notes from more than one hospital, rather than expanding the corpus with texts from a single institution, leads to better performance (83).

Other works create classifiers by exploiting modern DL architectures, such as CNNs, LSTMs, or RNNs. These models have been applied to classify free text reports from neuroimaging and magnetic resonance imaging (MRI) showing promising results (84, 85). In Tanana et al. (86) modern DL was used to identify sentiments in interactions between therapists and clients. Using a similar approach, Bacchi et al. (87) implemented a DL architecture to classify the causes of transient ischemic attacks from free medical texts. In all these efforts, the main limitations have always been related to DL architectures, as they require a huge amount of data to be trained.

An intrinsic limitation of NLP models based on feature-based extraction is that they do not take into account the context of words. Sarzynska-Wawer et al. (88) experienced this when they used the embeddings from language models (ELMo) approach (89) to represent interviews with patients suffering from schizophrenia and healthy individuals. Similar to BERT, ELMo is trained on a massive corpus to predict the next word in a sequence of words. This allows it to represent each token in an appropriate way, while retaining information about its context. This approach is more flexible compared to the feature-based approach, and provide solid performance.

### Natural language processing for data inference

The third class of papers is related to inference, which generally means that starting from the results of the NLP algorithm, some insights are obtained. In some cases, the models predict the patient’s disposition, while in others they are used to identify candidates for a particular treatment. Finally, some papers present models for the automatic analysis of specific pathologies.

One of the main challenges in predicting patient disposition is the limited nature of medical data, especially when working with DL architectures. This problem can be solved by using a medical tool developed for this purpose or by implementing a traditional ML model, that requires much less data. An example of the first solution is presented by Segev et al. (90), who used a clinical tool similar to Bio-YODIE to distinguish between side-effects of Clozapine (an antipsychotic drug) and myocarditis symptoms by analyzing case notes. Other examples are presented by Zhang et al. (91) and Funk et al. (92), who developed MetaPred, a framework for clinical risk prediction from longitudinal EHRs, and a framework to support automated analysis of text data in digital health interventions, respectively. The second approach is to use traditional classifiers, possibly in combination with one of the previously presented medical NLP tools. As already mentioned, these models have the advantage of requiring far less data to perform well compared to DL architectures. Examples of this approach are presented by Klang et al. (93), Ahuja et al. (94), and Irving et al. (95). The first developed an XGBoost model to predict admission to the intensive care unit within 30 min of emergency department (ED) arrival using free-text and tabular data, with good discriminatory performance. The second used a solution that combines both approaches: this study presents a model based on cTAKES for predicting relapse risk in multiple sclerosis, based on an L1-regularized logistic regression (LASSO) architecture. The third study used a similar architecture to identify individuals at risk for psychosis. When a large corpus is available, DL models are also a viable approach. This is the case with Tahayori et al. (96), who developed a BERT model to predict patient disposition based on triage notes in ED. This algorithm was specifically trained on free-text triage notes only, without considering other influential parameters such as age or vital signs; nevertheless, the performance of the approach is still robust.

Other works focus on a very crucial topic: identifying the best candidates for a given treatment. A reliable candidate selection algorithm could help overcome several limitations that have been demonstrated in the standard procedures used today: the benefits of timeliness of intervention, with better outcomes associated with earlier treatment (97); racial disparities in the use of some surgeries (98); the lack of a unifying pathophysiology (Iverson) (99). Dai et al. (100) tackled this problem from a generic standpoint, demonstrating that cohort selection of longitudinal patient records can be formulated as a multiple instance learning task. Candidate identification can be applied to very different medical topics: surgical intervention for drug-resistant pediatric epilepsy (101), risk factors for pediatric post-concussion symptoms (102), error reduction in determining eligibility for intravenous thrombolytic therapy (103).

On a slightly different topic, Wissel et al. (104) investigated a very sensitive matter, i.e., bias in ML algorithms trained on physician notes. This work assumed racial disparities in the use of epilepsy surgery. The authors developed an NLP algorithm and demonstrated that candidacy for epilepsy surgery was not biased by patient demographic information.

All the above-mentioned papers showed that free-text data from EHR are a valuable source of insight, and processing them with the proper tools could help clinicians to take decisions faster and more reliably.

The final task addressed in this category of articles was automatic analysis of specific pathologies. This is a broad topic, but the main goals were to exploit NLP to get an early (105, 106) and/or more accurate diagnosis (107) or to cope with the shortage of medical staff (108). With a similar approach, NLP can also be exploited to infer data for psychiatric conditions, such as suicidal thoughts in epilepsy subjects (109), depression (110), and obsessive-compulsive disorder (111).

All of the aforementioned works come to the same conclusion: properly trained NLP models for pathology analysis could be useful in conducting efficient medical practice. Of course, these tools have also their limitations. The most significant one is the fact that a model trained on a single hospital’s data may have a significant bias, and thus cannot be generalized when working with new data unless it is re-trained from scratch.

## Performance data comparison

This section presents the results of the 65 papers fully analyzed in this review. Not all of them had the same metrics, so it was not trivial to compare them. Most of these papers did not use a common reference dataset for the evaluation, which, as addressed in the Discussion, makes it difficult to compare the results. However, the most commonly used metric for each category was selected and reported.

The first class of works presented in this review is NLP for IE. The performance of these models is evaluated using typical statistical metrics of ML algorithms, such as: accuracy, precision, recall, F1-score (nomenclature and formulas are listed in Table 3). The most widely used parameter in the reported works was F1-score (harmonized average of recall and precision) which is often used in the NLP field as an overall measure of system performance. The performance of papers that adopt a traditional ML approach are presented in Table 4, while DL models are presented in Table 5.

**TABLE 3:** Performance measures.

Measure	Synonymous	Formula
Precision	Positive predictive value	TP/(TP + FP)
Recall	Sensitivity, true positive rate (TPR)	TP/(TP + FN)
F1-score	F-measure, F-score	2. (P. R)/(P + R)
Accuracy	–	(TP + TN)/(TP + FP + TN + FN)
False positive rate	False alarm ratio	FP/(FP + TN)
Area under ROC curve	AUC, AUROC, C-statistics	Trade-off between TPR and FPR. AUC = 1: perfect classifier, AUC = 0.5: random classifier

TP, true positive; TN, true negative; FP, false positive; FN, false negative; P, precision; R, recall; TPR, true positive rate; FPR, false positive rate.

**TABLE 4:** Comparison of NLP performance for information extraction papers via traditional ML models.

Author	Task	NLP model	Embeddings and Corpus	F1-score [%]
Savova et al. (42)	Development of cTAKES, tool for IE of EHR	Rule-based	PTB corpus (123): ∼910 k tokens	95.3
			GENIA corpus (124): ∼400 k words	93.2
			Mayo Clinic EHR: ∼100 k tokens	92.4
Fonferko-Shadrach et al. (51)	IE on epilepsy clinical texts	Rule-based	200 unstructured clinic letters	86.1

NLP, natural language processing; cTAKES, clinical text analysis and knowledge extraction system; IE, information extraction; EHR, electronic health record; PTB, penn treebank.

**TABLE 5:** Comparison of NLP performance for information extraction papers via DL models.

Author	Task	NLP model	Embeddings and Corpus	F1-score [%]
Lopes et al. (55)	NER pipeline for Portuguese EHR	LASSO	i2b2 corpus 2010: ∼1,500 EHR (https://www.i2b2.org/NLP/DataSets/)	83
Weng et al. (64)	IE pipeline built on cTAKES by using annotated texts	cTAKES + DL	iDASH corpus: 431 EHR (64)	84.5
			Massachusetts General Hospital corpus: ∼90 k EHR	87
Yu et al. (56)	NER pipeline by using unannotated texts	BERT-based + CNN-based	BioWordVec: ∼2.3 M words (https://github.com/ncbi-nlp/BioWordVec)	91.4
Kraljevic et al. (47)	Development of MedCAT, tool for IE of EHR	BERT-based	Self-supervised train: ∼ 8.8B words Fine-tune: ∼6 k annotated examples	94
Vaci et al. (61)	Extract data on individuals with depression from EHR	LSTM	1.8 M EHRs from UN-CRIS Database (61)	69

NLP, natural language processing; NER, named entity recognition; LASSO, L_1_-regularized logistic regression; i2b2, informatics for integrating biology and the bedside; IE, information extraction; cTAKES, clinical text analysis and knowledge extraction system; DL, deep learning; BERT, bidirectional encoder representations from transformers; CNN, convolutional neural network; MedCAT, medical concept annotation toolkit; EHR, electronic health record; PTB, penn treebank; LSTM, long short-term memory.

As far as the NLP for classification tools are concerned, the metric used for the evaluation is usually the area under the curve (AUC), as visible in Table 6, referred to papers that analyze texts produced by patients. A different approach uses notes from clinicians to classify patients, as reported in Table 7.

**TABLE 6:** Comparison of NLP performance for classification of texts produced by patients.

Author	Classification	NLP model	Embeddings and Corpus	AUC
Takano et al. (72)	Study 1: Specific vs. non-specific memory	SVM	Study 1: ∼12,400 EHR	0.92
	Study 2: Novel memories		Study 2/3: ∼8,500 EHR	0.89
Clark et al. (65)	MCI vs. AD	RF + SVM + naïve Bayes + Multilayer perceptrons	Fluency scores from ∼150 patients	0.872
Wang et al. (68)	COVID-19 Twitter data analysis	RF*	50 M Tweets	0.966
Yu et al. (71)	Negative life events into categories	SVM	Unlabeled corpus: 5,000 forum posts Labeled corpus: 2,856 sentences from ISP-D database of PsychPark (125)	0.897

NLP, natural language processing; AUC, area under curve; SVM, support vector machine; RF, random forest; EHR, electronic health record; MCI, mild cognitive impairment; AD, Alzheimer’s Disease. *Only best classifier performance is reported.

**TABLE 7:** Comparison of NLP performance for classification from clinicians’ notes.

Author	Task	NLP model	Embeddings and Corpus	AUC
Xia et al. (77)	Identify patients with complex neurological disorder	cTAKES-based	Train corpus: ∼600 clinical notes Test corpus: ∼500 clinical notes	0.96
Wissel et al. (104)	Identify candidates for epilepsy surgery	Multiple linear regression	Train corpus: ∼1,100 clinical notes Test corpus: ∼8,340 clinical notes	0.9
Heo et al. (85)	Prediction of stroke outcomes	CNN + LSTM + Multilayer perceptron	Train corpus: ∼1,300 clinical notes Test corpus: ∼500 clinical notes	0.81
Lineback et al. (78)	Prediction of 30-day readmission after stroke	Logistic regression + naïve Bayes + SVM + RF + Gradient boosting + XGBoost	Train corpus: ∼2,300 clinical notes Test corpus: ∼550 clinical notes	0.64
Lin et al. (81)	Identify UAU in hospitalized patients	Logistic regression	Train corpus: ∼58 k clinical notes	0.91
Bacchi et al. (87)	Prediction of cause of TIA-like presentations	RNN + CNN	Corpus: 2,201 clinical notes (∼150 words each)	0.88

NLP, natural language processing; AUC, area under curve; cTAKES, clinical text analysis and knowledge extraction system; CNN, convolutional neural network; LSTM, long short-term memory; SVM, support vector machine; RF, random forest; UAU, unhealthy alcohol use; ISP-D, internet-based self-assessment program for depression.

The final class of works presented in this review is NLP for data inference. This class of papers is broad and includes different types of works. Table 8 reports the results of the first sub-class, i.e., the model for predicting the disposition of patients. The second subclass is related to the identification of the best candidates for a specific treatment, and the statistical parameter for evaluation is the F1-score (Table 9). The last subclass is automatic analysis of specific pathologies (Table 10) evaluated using the F1-score.

**TABLE 8:** Comparison of NLP performance for predicting patient disposition.

Author	Prediction	NLP model	Embeddings and Corpus	AUC
Tahayori et al. (96)	Patients’ disposition from triage notes	BERT-based	∼250 k EHR	0.88
Ahuja et al. (94)	Relapse risk for multiple sclerosis	LASSO	Train corpus: ∼1,400 clinical notes Validation corpus: ∼200 clinical notes	0.71
Zhang et al. (91)	Clinical risk	CNN + LSTM	2.5 M patients’ EHR	0.85
Klang et al. (93)	Neuroscience ICU admission	XGBoost	∼412 k patients’ EHR	0.93

NLP, natural language processing; AUC, area under curve; BERT, bidirectional encoder representations from transformers; LASSO, L_1_-regularized logistic regression; CNN, convolutional neural network; RNN, recurrent neural network; LSTM, long short-term memory; EHR, electronic health record; ICU, intensive care unit; TIA, transient ischemic attack.

**TABLE 9:** Comparison of NLP performance for identification of the best candidates for a specific treatment.

Author	Identification	NLP model	Embeddings and Corpus	F1-score [%]
Cohen et al. (101)	Candidates for surgery for drug-resistant epilepsy	Naïve Bayes	∼6,300 patients’ EHR	82
Sung et al. (103)	Intravenous thrombolytic therapy candidates	MetaMap-based	234 clinical notes	98.6

NLP, natural language processing; EHR, electronic health record.

**TABLE 10:** Comparison of NLP performance for analysis of specific pathologies.

Author	Analyzed pathology	NLP model	Embeddings and Corpus	F1-score [%]
Castro et al. (107)	Cerebral aneurysms	cTAKES-based + LASSO	Train corpus: ∼300 clinical notes (manually annotated) Validation corpus: ∼17 k clinical notes	84
Katsuki et al. (108)	Primary headache	DL-based	Test corpus: ∼850 clinical notes	63.5

NLP, natural language processing; cTAKES, clinical text analysis and knowledge extraction system; LASSO, L_1_-regularized logistic regression; DL, deep learning.

**FIGURE 3:**
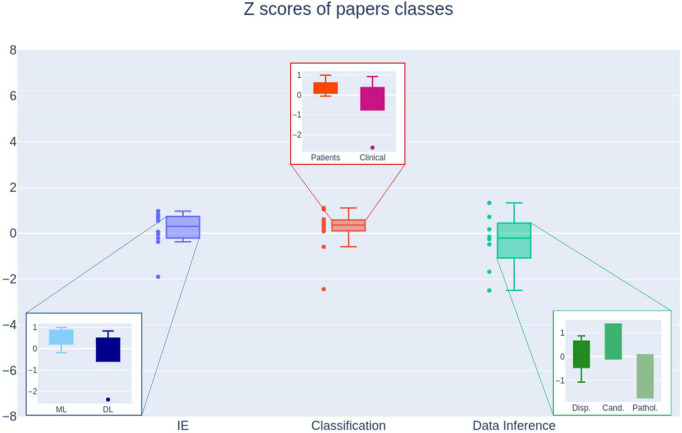
Performance box plot of the NLP methods representing the medians, interquartile ranges, outliers. IE, information extraction; ML, traditional machine learning; DL, deep learning; Patients, patients texts; Clinical, clinical notes; Disp., patients’ disposition; Cand., best candidates; Pathol., specific pathologies.

Overall, the performance of NLP applications appeared to be high, with an average F1-score and an AUC above 85%. It has to be highlighted once again that the performance of reported papers is measured on very specific datasets, and thus a direct comparison is not feasible. Because the heterogeneity, in order to get a quantitative index, we also derived a composite measure in the form of Z-scores to better compare and understand the performance of the NLP models and their different classes as a whole (Figure 3). No statistical differences were found in the unbiased comparison (*p*-value > 0.05, calculated with Kruskal–Wallis test).

## Discussion

Our review shows that NLP is used for many different purposes in neuroscience and psychiatry. In the last years, the interest of the scientific community studying NLP has grown exponentially, and the same is true for the medical community. The major factors in this growth have been: (i) the increasing adoption of EHRs, (ii) the urge to find an automatic way to analyze such EHRs, (iii) the proliferation of ML/DL models in healthcare.

Entering patient data into clinical notes is time-consuming for physicians: about 35% of their time, according to Joukes et al. (112). Even though a structured text provides faster access to information than a free-text report, the time required for this operation is often too high, leading to underusing EHRs data because of their hard accessibility. This means that a lot of useful information is often discarded simply because it would take too much effort to extract it. This effort could be partially avoided by using NLP models, as they provide a way to find and extract relevant insights from data hidden in medical records.

The first significant turning point in the development of NLP was the transition from statistical models to ML. Statistical models are typically rule-based, meaning that if a text does not conform to the rules, the system performs very poorly. ML models are more versatile, can address non-linear problems and are therefore more usable. The second turning point was the introduction of DL models. The most recent ones can take into account the context of words. This makes them even more flexible than the classical ML approaches and able to analyze completely unstructured texts.

As mentioned earlier, DL models require an enormous amount of data to be properly trained. Fortunately, the NLP community is often open-source; this means that many pre-trained models are freely accessible and usable. Nevertheless, the data needed to fine-tune NLP models must be labeled, which means that some experts in the field must manually annotate the fine-tuning corpus. Thus, the data needed to fine-tune NER models must be fed with text containing the appropriate entities. For example, the sentence “*He has cognitive problems due to Alzheimer’s disease, and after examining his brain, the doctor prescribed him Donepezil*” should be annotated with the following entities:

1. “*Alzheimer’s disease*” = PATHOLOGY
2. “*brain*” = ANATOMY
3. “*cognitive problems*” = MEDICAL_CONDITION
4. “*Donepezil*” = DRUG

This operation is lengthy and tedious, but it is often fundamental to increase performance. Despite the difficulty of this process, once a model is properly trained the advantages of its usage are obvious. It can be used for a variety of tasks. Some examples include: data entry into databases [by converting unstructured clinical information into structured data, Futrelle et al. (113)], extract relevant information from patient records to support clinical decisions (e.g., properly selecting patients for an innovative treatment to maximize the chances of success, recruiting participants for specific clinical trials, …), categorize/stratify clinical documents, summarize clinical information stored in long EHRs, and search and classify clinical terms into ICD-10/SNOMED diagnosis codes (47). More recently, NLP has been used to develop conversational bots to provide initial instructions to patients in critical situations, e.g., during the COVID-19 pandemic infection peaks when primary care physicians had to answer dozens/hundreds of calls daily (114). Clearly, the role of the physician is essential for the adoption of NLP in clinical practice. Their expertise would make them the perfect testers of NLP tools (e.g., QABot, NER, information grouping/retrieving, summarization, …), and the final evaluators in terms of user-friendliness, utility and usability of such systems.

As a final remark, none of the three macro-categories presented in the comparison Section shows dominant performance respect to the others. This proves that NLP is flexible and can be applied to every task with similar results.

It has to be highlighted that, although NLP has a great potential, some issues have to be taken into account.

### Language issues

The first issue is the strong asymmetry between English and non-English availability of corpora and models. Of the 65 papers in the quantitative search, five developed non-English based models, representing 7.7% of the total. Moreover, there are ∼5,000 English models in the HF repository, but only five Chinese, 51 Hindi, and 400 Spanish, even though these are the second, third, and fourth most spoken languages in the world, respectively. This can be problematic, especially if one plans to use DL architectures in an NLP model, as these require a massive amount of data to be trained (e.g., BERT required more than 3 billion words for pre-training). This problem can be partially mitigated by the availability of multi-language models (there are dozens of them available in the HF public repository). Starting from such models, the user should only need to fine-tune them, which requires a much smaller amount of data (e.g., fine-tuning a BERT model requires tens to hundreds of thousands of words). Another approach could be automatic translation of English corpora, but this inevitably introduces bias and the effectiveness of this approach has yet to be fully demonstrated.

### Annotation problems

The typical workflow is to start from a pre-trained model and fine-tune it, thus exploiting a model that has a general understanding of the language. Fine-tuning, the operation that allows to specialize a generic pre-trained model to a specific task, requires much less data and computing power. However, data for this operation have to be labeled (in contrast to the unsupervised nature of pre-training). This means that, especially for very specific medical domains (e.g., neurology), labeling must be done by experts in that specific field, so it is not trivial to produce high quality labeled data.

### Standard terminology

Natural language processing systems can be very sensitive to the use of non-standard terminology. Because medical terms can be very specific and unusual, there is no guarantee that synonyms will be recognized as such. The most common standards associated with medical topics are the International Statistical Classification of Diseases and Related Health Problems (ICD), the Systemized Nomenclature of Medicine–Clinical Terms (SNOMED-CT), and the UMLS. A possible solution could be the use of a very large corpus containing different terms and acronyms used in an interchangeable way. Indeed, DL based model embeddings take into account the context of a word and different words used in a very similar context would likely have a similar representation. Another way to approach the problem would be to use a model that has been trained by taking into account issues related to nomenclature and ontologies, e.g., the aforementioned Umls-BERT.

### Training/test data not always comparable across studies

One possible source of performance degradation is that a model trained on corpora from a single hospital can cause bias in the model, making it very focused and unable to generalize (83). The best solution, if possible, is to include text from different medical institutions in the training corpus. This way, the model can benefit from different text representations and thus improve its generalization capabilities.

### Other possible issues

Another gap, when speaking about medical NLP, is that a universally recognized state-of-the-art model is still missing. This comes as no surprise, since the topic is still in its infancy. Moreover, implementing a NLP pipeline is a complex and burdensome task, that requires both programming skills and medical knowledge. NLP tools need to be developed through a synergy between IT people for model creation and medical staff for the annotation part. It is important to remember that these tools, like any ML model, are only as good as the quality of the data they are trained with. Another limitation, related in part to this issue, is the lack of a standardized, universally accepted medical data set for model evaluation. From this review, it appears that almost all the papers presented used their own data to develop and test models. This may introduce bias when comparing different models and make the comparison difficult. Nonetheless, in the last years international organizations have made efforts to overcome this gap. One of them is the Conference and Labs of the Evaluation Forums (CLEF),^9^ whose main mission is to promote research of information access systems with an emphasis on multilingualism and multimodality. Such organizations promote workshops and/or competitions, where research teams all over the world can validate their models in an unbiased and scientific way. Some of the most known events are:

1. BioASQ^10^ : sponsored by Google and the European Union, BioASQ organizes challenges on biomedical semantic indexing, including text classification, information retrieval, QA from texts and structured data, multi-document summarization and many other areas;
2. n2c2^11^ : organized by Harvard Medical School, it is an annual context where participants have to develop models able to perform biomedical NLP tasks (e.g., NER, IE, event detection);
3. The International Workshop on Semantic Evaluation (SemEval): a series of international NLP research workshops whose mission is to advance the current state-of-the-art in semantic analysis and to help create high-quality annotated datasets in a range of increasingly challenging problems in natural language semantics.

In addition, only a few of the analyzed papers exploited DL architectures. Most of the works in this review used NLP only as a tool to get features for traditional classifiers (SVM, RF, …). While this is the only possible approach in some situations (e.g., when the training corpus is small and therefore cannot be used for DL architectures), DL models have shown better results for classification tasks, so their approach may lead to more robust models in the future.

Finally, another limitation, especially for medical personnel, is the lack of plug-and-play NLP tools. This restriction is even more heavy for non-English speakers. Although there are many tools available to perform NLP analysis and some easy to use open-source libraries, this still could represent a barrier for people without a solid IT background. Wider availability of user-friendly NLP tools could lead to greater interest in the topic, increasing the competition and the future quality of studies in this sector.

### Limitations

This review has a number of limitations. The most significant one is the fact that it analyses only articles from the first quartile rank. While this ensures that the papers examined are of high-quality, it could lead to the *a priori* exclusion of effective tools. Another limitation is that speech-related works were not included. This topic has become more important in recent years, in the context of the increasingly prevalence of voice assistants. However, as explicitly stated in the introduction, the research question of this work was to analyze tools that manipulate clinical documents, and therefore this class of papers was excluded.

## Conclusion

Natural language processing algorithms hold promise for helping physicians to get insights from unstructured texts such as medical reports, clinical research form EHR data, and more. Advanced NLP techniques will enable machines to understand, classify, summarize, and generate text to automate medical linguistic tasks. There are many tools that make NLP accessible and publicly available for medical areas, such as: HF, spaCy, and other well-supplied resources. Open-source libraries are flexible and allow developers to fully customize NLP resources. So far, they are not fully cost-effective and require to spend time to create and train *ad hoc* NLP models before they can be used in the medical fields. The performance of NLP applications is generally high, but few systems are actually used in routine clinical care or research. The establishment of minimum requirements, further standardization, and external validation will likely soon increase the prevalence of NLP applications in neuroscience and psychiatry.

## Data availability statement

The original contributions presented in this study are included in the article, further inquiries can be directed to the corresponding author.

## Author contributions

CC: methodology, investigation, formal analysis, conceptualization, visualization, and writing. GA and DS: review and editing. AR: methodology, conceptualization, writing, and supervision. All authors contributed to the article and approved the submitted version.

## Footnotes

1
https://wenxin.baidu.com/

2
https://www.i2b2.org/

3
https://www.nltk.org/

4
https://spacy.io/

5
http://hdl.handle.net/1721.1/119754

6
https://gate.ac.uk/

7
https://au.reachout.com/

8
http://www.jtf.org.tw

9
http://www.clef-initiative.eu/

10
http://bioasq.org/

11
https://portal.dbmi.hms.harvard.edu/data-challenges/
